# Rapid Clinical Recovery in Streptococcal Toxic Shock Syndrome With Intravenous Immunoglobulin (IVIG): A Case Report

**DOI:** 10.7759/cureus.94928

**Published:** 2025-10-19

**Authors:** Suhad Jalodi, Muhammad Naseem, Syed Abubacker, Aima Hameed, Aymen Rasool

**Affiliations:** 1 Internal Medicine, Kettering General Hospital, Kettering, GBR; 2 General Internal Medicine, Kettering General Hospital, Kettering, GBR; 3 Acute Medicine, Kettering General Hospital, Kettering, GBR

**Keywords:** intravenous immunoglobulin (ivig), sepsis and septic shock, streptococcal toxic shock syndrome (stss), streptococcus pyogenes infections, stss

## Abstract

Streptococcal toxic shock syndrome (STSS) is a rare but life-threatening condition, with an estimated incidence of 1-3 cases per 100,000 population annually and reported mortality rates exceeding 30% and reaching up to 44% in some series. It is characterized by rapid systemic deterioration due to toxin-producing *Streptococcus pyogenes*. Prompt recognition and escalation of therapy, including the use of adjunctive intravenous immunoglobulin (IVIG), are critical to improving outcomes. We report the case of a previously healthy 37-year-old man who presented with high-grade fever, hypotension, and a widespread erythematous rash. Initial antibiotic therapy targeting skin and soft tissue infection yielded a limited response. Culture from an ulcerated lesion confirmed beta-hemolytic *Streptococcus*. Despite broad-spectrum antibiotics and intensive care support, the patient continued to deteriorate. A diagnosis of STSS was made, and IVIG was administered with rapid clinical improvement. This case highlights the importance of early diagnosis, multidisciplinary management, and the potential role of IVIG in severe STSS.

## Introduction

Streptococcal toxic shock syndrome (STSS) is a rare but life-threatening toxin-mediated condition caused by invasive *Streptococcus pyogenes *(group A *Streptococcus*), with an estimated incidence of 2-4 cases per 100,000 people per year [1,2]. Despite its rarity, STSS carries a high mortality rate exceeding 25-30%, often due to the rapid onset of shock and multiorgan dysfunction [3].

The pathophysiology of STSS is primarily driven by pyrogenic exotoxins that act as superantigens, bypassing conventional antigen processing and directly activating large numbers of T lymphocytes [4]. This massive T-cell activation leads to excessive cytokine release, widespread endothelial damage, capillary leak, and subsequent circulatory collapse [5].

Clinically, the Centers for Disease Control and Prevention (CDC) defines STSS by the presence of hypotension, isolation of group A *Streptococcus *from a sterile or non-sterile site, and involvement of at least two organ systems, including renal, hepatic, hematologic, pulmonary, or cutaneous manifestations [6]. These diagnostic criteria underscore the rapid systemic deterioration that often characterizes the disease.

While the mainstay of treatment remains early antimicrobial therapy, source control, and organ support, recent evidence suggests a potential role for intravenous immunoglobulin (IVIG) in improving outcomes, particularly when used alongside clindamycin [7-10]. This case highlights the importance of early recognition and multidisciplinary management and the potential impact of adjunctive IVIG in the management of STSS.

## Case presentation

**Figure 1:**
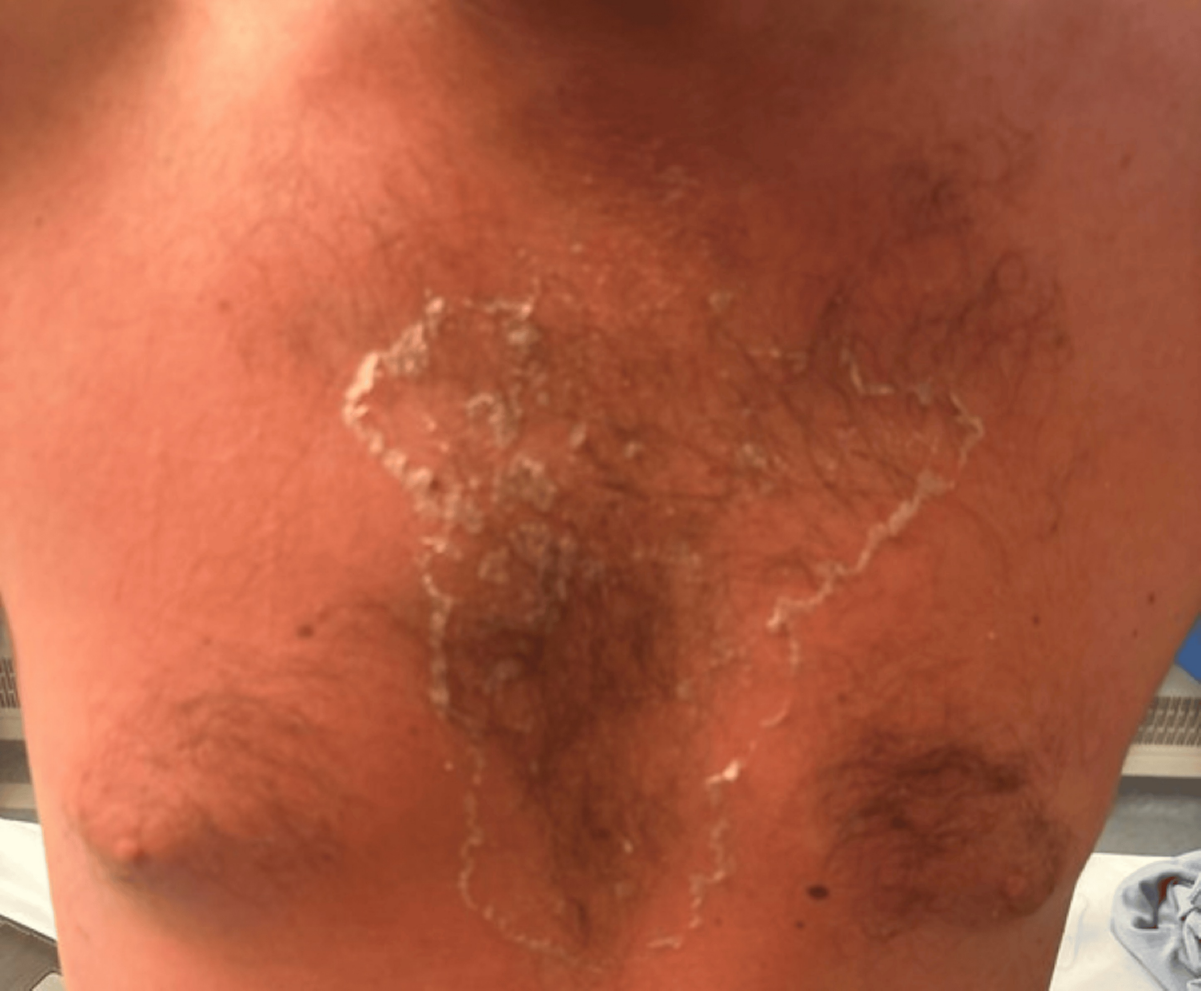
Rash across the chest

**Figure 2:**
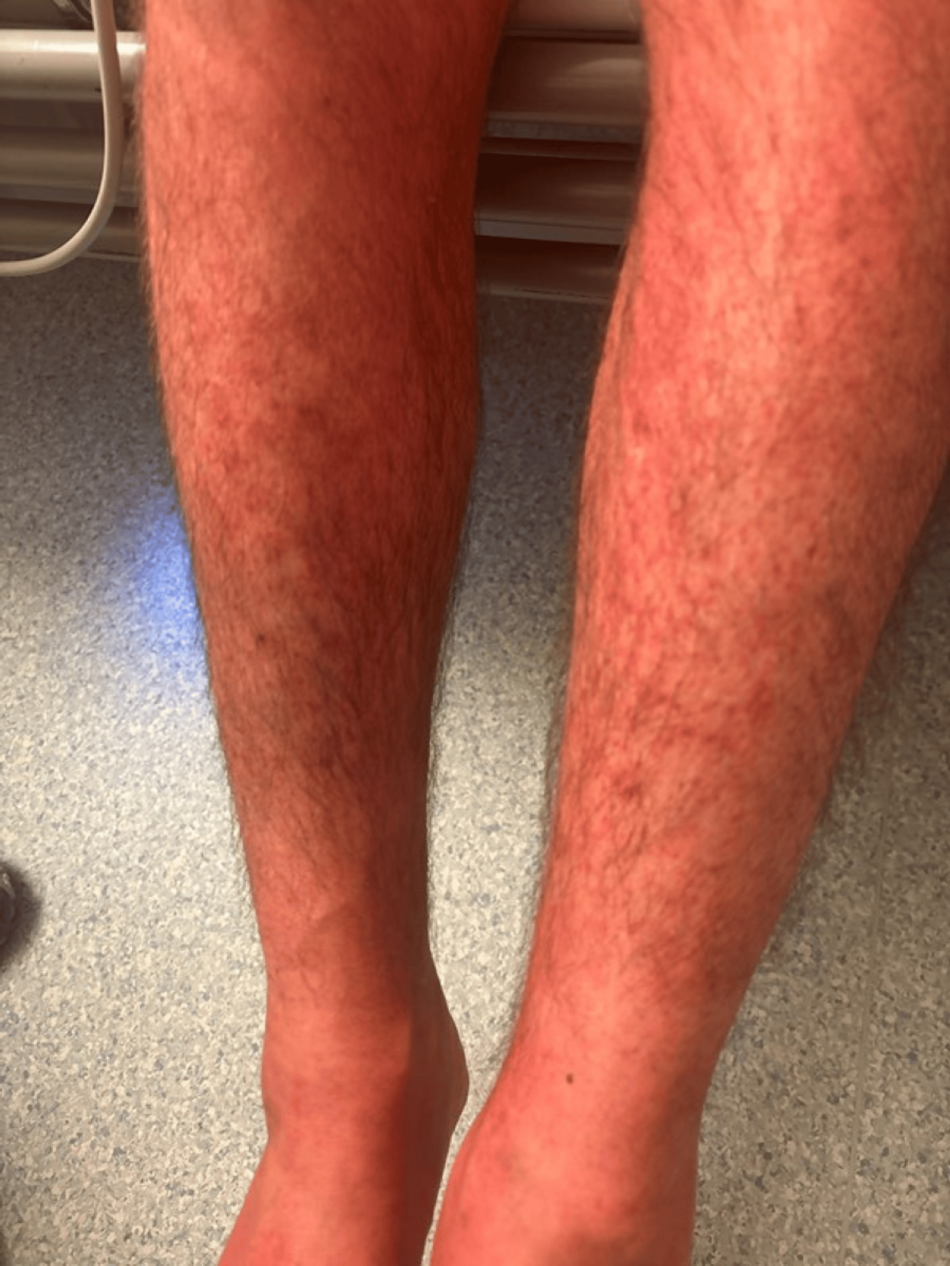
Rash on both legs (before treatment)

**Figure 3:**
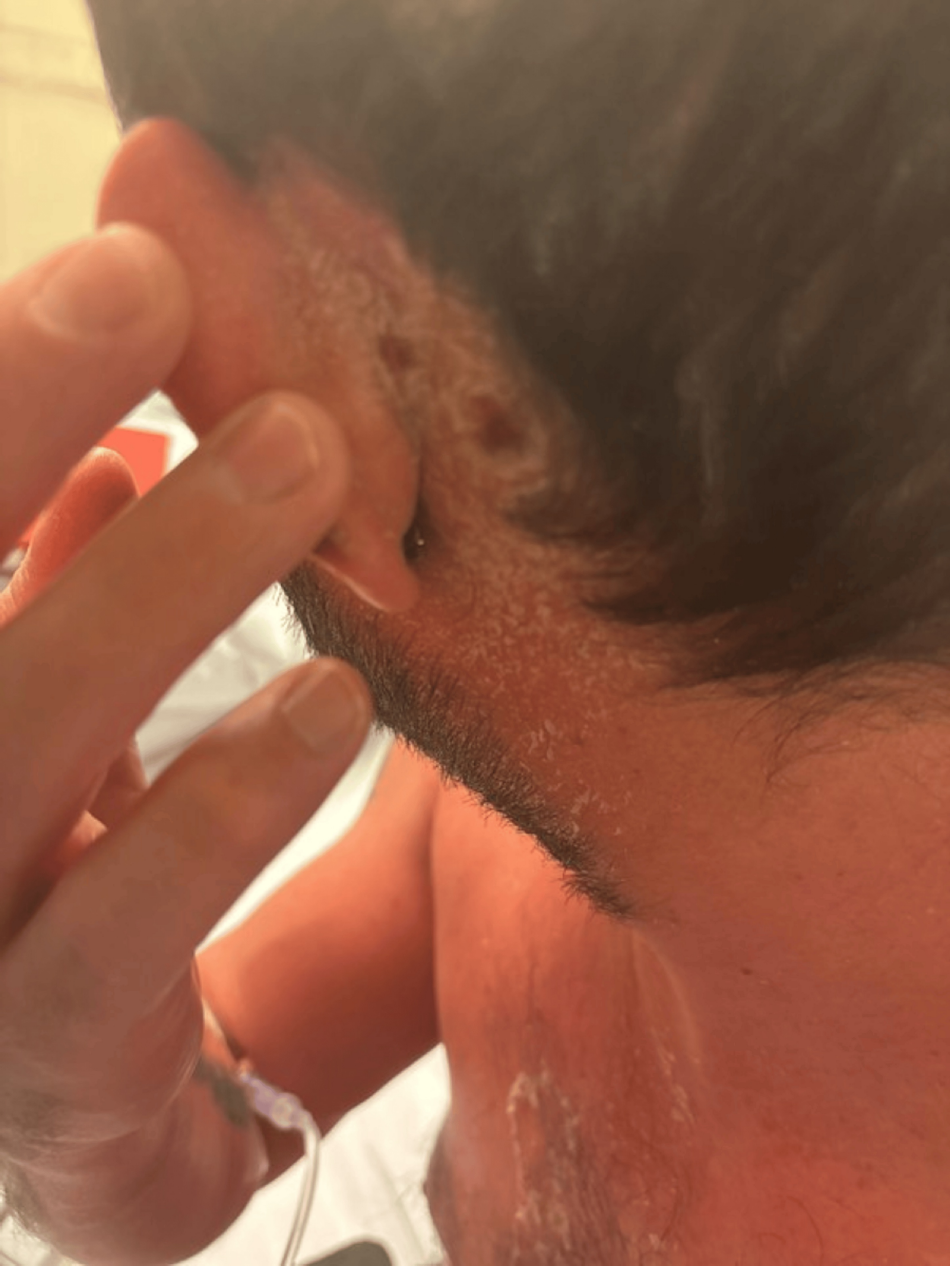
Ulcerative lesion behind the left ear

A 37-year-old previously healthy man presented to the emergency department with a 48-hour history of high-grade fever, hypotension, and a widespread non-blanching erythematous rash (Figures 1-2). He also reported malaise and dizziness. On examination, he was febrile (39.2°C), hypotensive (BP 85/50 mmHg), and tachycardic (HR 110 bpm). A 2×3 cm ulcerative lesion with surrounding erythema was noted below his left ear (Figure 3), the origin being traumatic.

Laboratory investigations revealed leukocytosis (WBC 14.1×10⁹/L), neutrophilia (13.0×10⁹/L), and markedly elevated C-reactive protein (CRP) (226 mg/L). Renal functions (sodium 123 mmol/L, creatine 114 umol/L, and estimated glomerular filtration rate (eGFR) 66.6) and liver function tests (bilirubin 22 umol/L, albumin 31 g/L, alanine aminotransferase (ALT) and alkaline phosphatase (ALP) normal) were slightly deranged on admission. Clotting profile was within normal limits. The patient was started on intravenous flucloxacillin (2 g every six hours) targeting a presumed skin and soft tissue infection.

**Figure 4:**
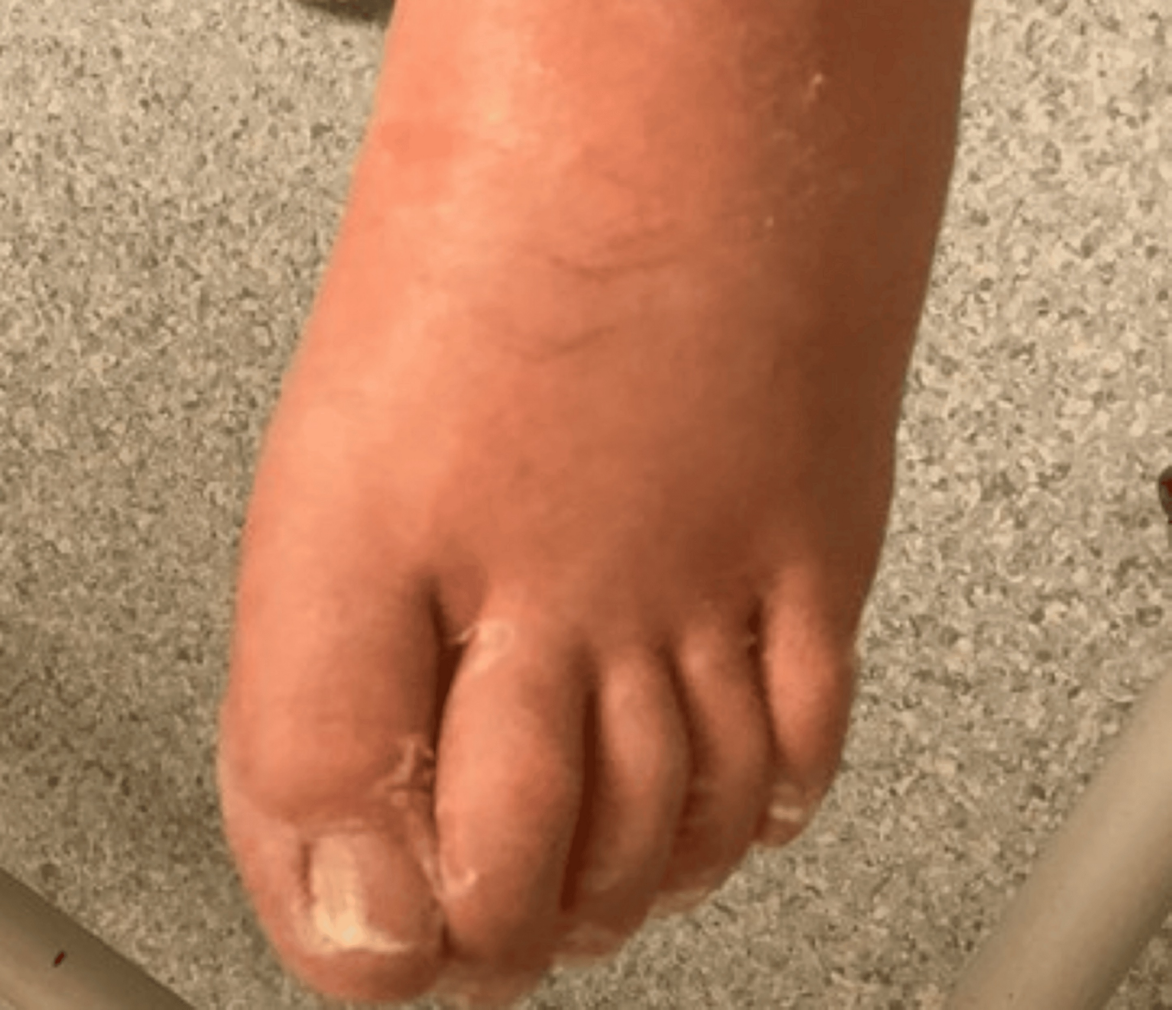
Oedema noted after fluid resuscitation

A wound swab from the ulcerative lesion cultured beta-hemolytic *Streptococcus*, sensitive to penicillin (Table 1). Despite appropriate antimicrobial coverage, he remained febrile (38.2°C) and hypotensive (BP 89/56 mmHg). Substantial fluid resuscitation was administered, totaling approximately 6-7 litres over a 24-hour period. Subsequently, the patient developed generalized oedema secondary to fluid overload (Figure 4). Despite this, renal function improved significantly, with an eGFR exceeding 90 mL/min/1.73 m².

**Table 1:** Swab culture and antibiotic susceptibility (May 14, 2025)

Specimen	Organism isolated	Antibiotic	Result
Swab skin	Beta-hemolytic group A *Streptococcus*	Penicillin	S
		Erythromycin	R
		Doxycycline	R
	Staphylococcus aureus	Penicillin	R
		Erythromycin	S
		Doxycycline	S
		Flucloxacillin	S

Ongoing clinical deterioration prompted the escalation of antibiotic therapy to intravenous piperacillin-tazobactam (4.5 g TDS) and clindamycin (1200 mg QDS initially for the first day and then 900 mg QDS for the remaining four days) for its anti-toxin properties. Dermatology and microbiology input supported a clinical diagnosis of STSS. Given ongoing deterioration despite antibiotic therapy and fluid resuscitation, a discussion was made with both microbiology and dermatology teams, and he was promptly commenced on IVIG (Kiovig 70 g administered for two days) as it was presumed to be a life-threatening condition.

**Figure 5:**
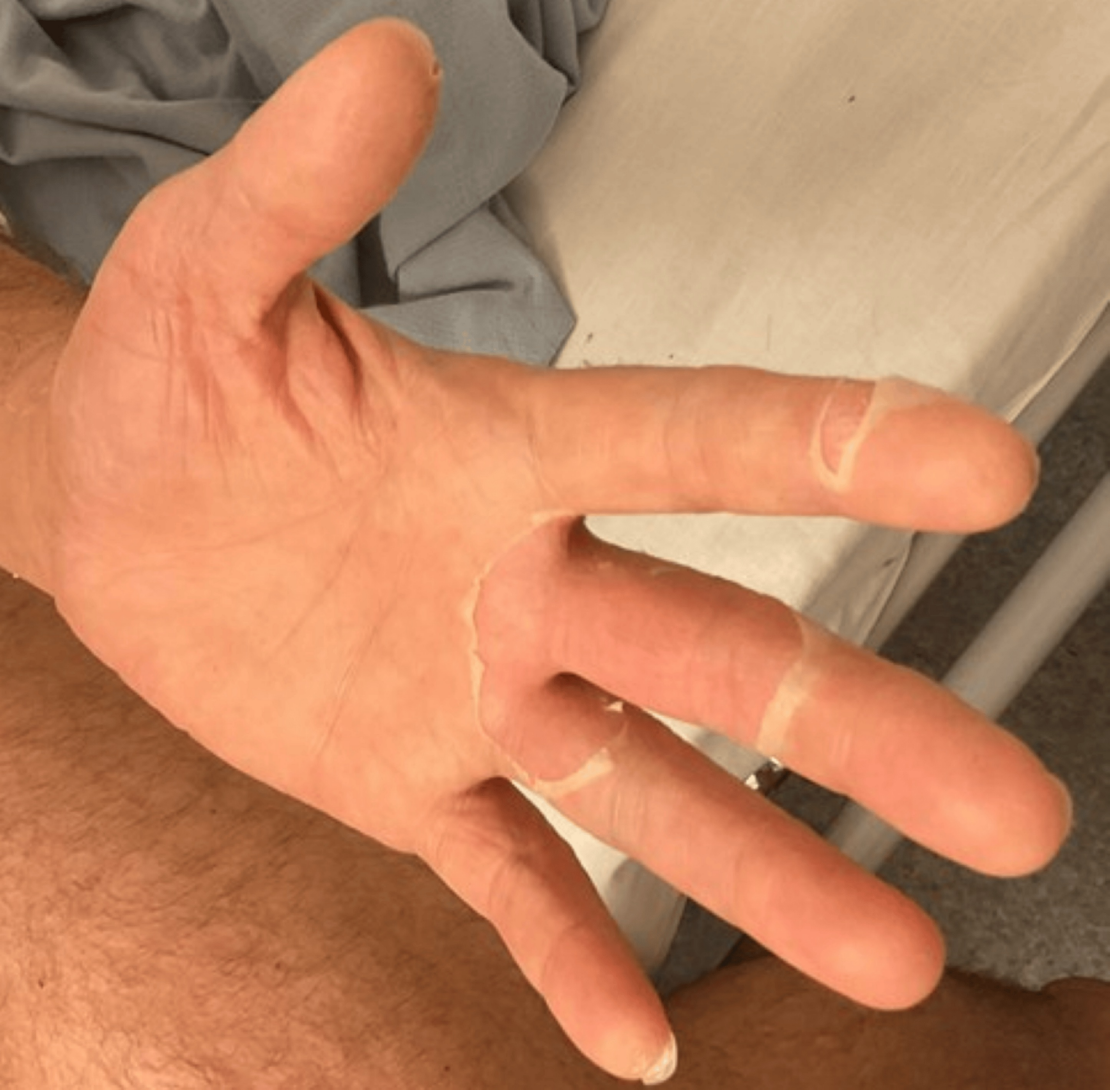
Desquamation of the skin following IVIG treatment IVIG: intravenous immunoglobulin

**Figure 6:**
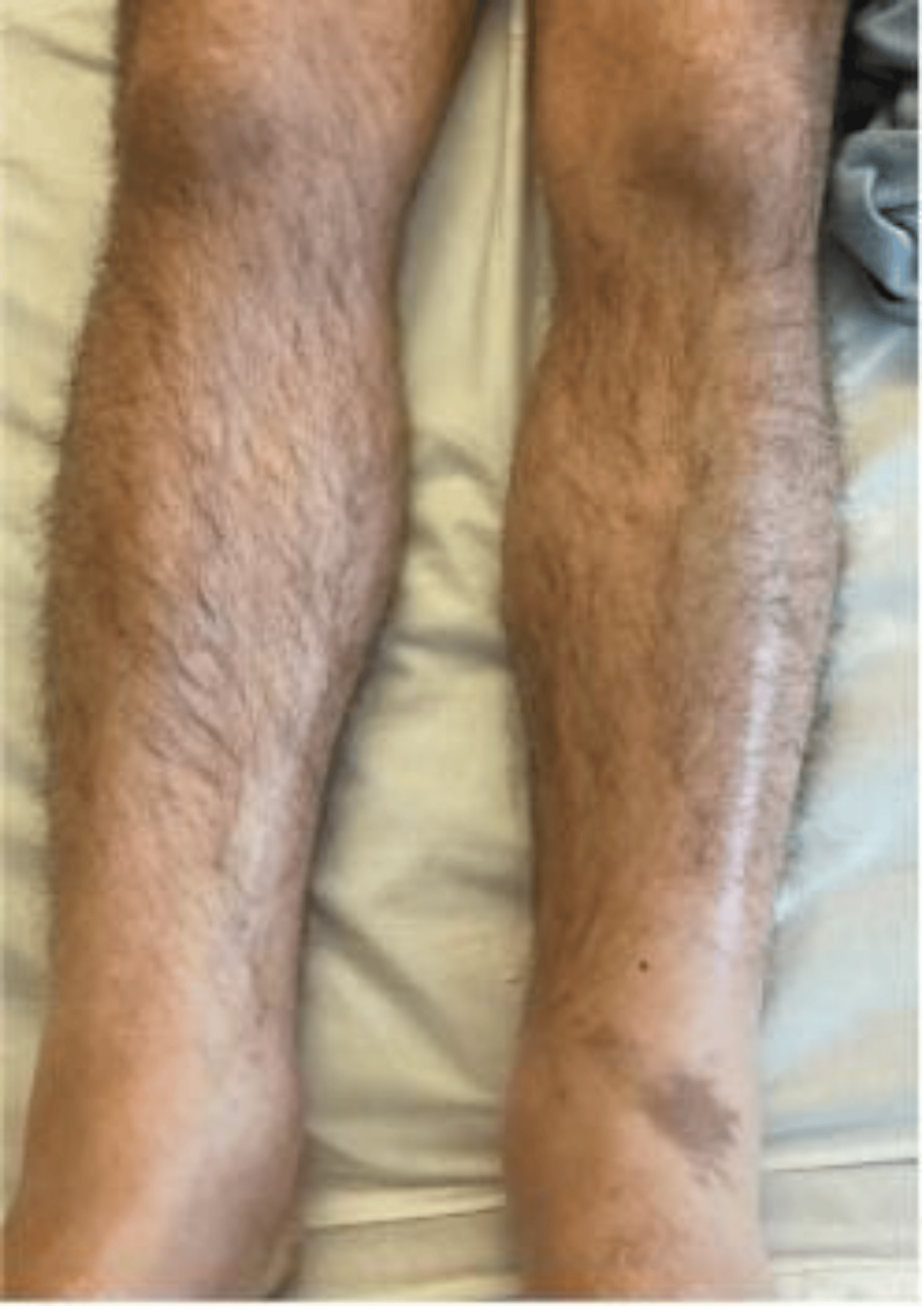
Resolution of rash over the legs after treatment with IVIG IVIG: intravenous immunoglobulin

Following IVIG therapy, the patient exhibited rapid clinical improvement. Fever resolved, blood pressure normalized without vasopressors, and the rash significantly improved (Figures 5-6). He was de-escalated to oral co-amoxiclav three days post-IVIG and discharged home in stable condition on day 4. He remained clinically well on outpatient follow-up.

## Discussion

STSS remains a rapidly progressive, life-threatening illness caused by *Streptococcus pyogenes*, with mortality often exceeding 25-30% despite optimal care [1-3]. Clinically, it typically presents with a sudden onset of high-grade fever, hypotension, and multiorgan dysfunction, frequently in association with a soft tissue focus of infection [7,8].

In our patient, the diagnosis of STSS was supported by persistent hypotension despite fluid resuscitation, high-grade fever, and markedly elevated inflammatory markers, together with a confirmed soft tissue infection on microbiological culture. These features, in combination with rapid clinical deterioration despite broad-spectrum antimicrobial therapy and aggressive fluid resuscitation, prompted escalation to adjunctive therapy.

The cornerstones of management are early recognition, prompt initiation of effective antimicrobials (including clindamycin for its antitoxin properties), aggressive hemodynamic support, and surgical source control where indicated [7,8].

Adjunctive IVIG has been proposed due to its potential to neutralize streptococcal superantigens and modulate the host inflammatory response [7,11]. Meta-analyses combining one randomized trial and several observational studies have shown a reduction in mortality from approximately 33.7% to 15.7% in clindamycin-treated patients who received IVIG [7]. A prospective cohort from Sweden demonstrated significantly improved 28-day survival with IVIG use (adjusted odds ratio 5.6) despite greater severity in the IVIG group [8]. Similarly, a Canadian study reported a survival rate of 67% in IVIG recipients compared to 34% in controls (odds ratio 8.1), alongside reductions in inflammatory cytokine responses [9].

Although the only randomized controlled trial was underpowered due to early termination, it did demonstrate improved organ failure scores and a trend toward lower mortality in the IVIG group [7]. The proposed mechanism includes neutralization of streptococcal superantigens and modulation of the host inflammatory response [4,7].

However, a recent large Japanese observational study involving a broader cohort of invasive group A streptococcal infections reported no significant mortality benefit with IVIG, though STSS-specific subgroup analysis was limited [11]. This discrepancy highlights the need for larger, STSS-focused randomized trials.

**Figure 7:**
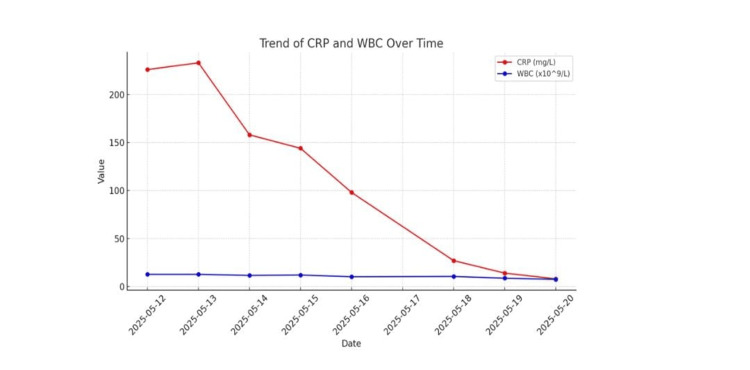
Trend of CRP and WBC over time CRP: C-reactive protein; WBC: white blood cell

In our patient, the decision to escalate to IVIG was made after persistent fever, hypotension, and inflammatory marker elevation despite optimal antimicrobial therapy and fluid resuscitation. The rapid clinical improvement and marked biochemical response, demonstrated by the trends in CRP and WBC (Table 2 and Figure 7), support its potential role as an adjunctive therapy in refractory STSS.

**Table 2:** Trend of inflammatory markers (CRP and WBC) during hospital admission. A marked decline in CRP and WBC was observed following the initiation of IVIG therapy on day 4, indicating the rapid resolution of systemic inflammation CRP: C-reactive protein; WBC: white blood cell; IVIG: intravenous immunoglobulin

Date	CRP (mg/L)	Reference range CRP (mg/L)	WBC (×10⁹/L)	Reference range WBC (×10⁹/L)
12/05/2025	226	<5	12.7	4.0-11.0
13/05/2025	233	<5	12.7	4.0-11.0
14/05/2025	158	<5	11.6	4.0-11.0
15/05/2025	144	<5	12.0	4.0-11.0
16/05/2025	98	<5	10.2	4.0-11.0
18/05/2025	27	<5	10.5	4.0-11.0
19/05/2025	14	<5	8.7	4.0-11.0
20/05/2025	8	<5	7.5	4.0-11.0

## Conclusions

This case underscores the importance of early recognition and aggressive multidisciplinary management of STSS. Clinicians should maintain a high index of suspicion for STSS in patients with rapidly progressive sepsis and confirmed streptococcal infection, even in previously healthy individuals. IVIG may serve as a valuable adjunct in cases where standard antimicrobial therapy and supportive care are insufficient.
